# Use of Physiologically-Based Pharmacokinetic Modeling to Simulate the Profiles of 3-Hydroxybenzo(a)pyrene in Workers Exposed to Polycyclic Aromatic Hydrocarbons

**DOI:** 10.1371/journal.pone.0102570

**Published:** 2014-07-17

**Authors:** Roberto Heredia Ortiz, Anne Maître, Damien Barbeau, Michel Lafontaine, Michèle Bouchard

**Affiliations:** 1 Département de santé environnementale et santé au travail, Chaire d’analyse et de gestion des risques toxicologiques and Institut de recherche en santé publique de l’Université de Montréal, Université de Montréal, Montréal, Québec, Canada; 2 Equipe environnement et prédiction de la santé des populations, Laboratoire TIMC (UMR 5525), CHU de Grenoble, Université Joseph Fourier, La Tronche, France; 3 Institut national de recherche et de sécurité, Vandoeuvre, France; Northwestern University Feinberg School of Medicine, United States of America

## Abstract

Biomathematical modeling has become an important tool to assess xenobiotic exposure in humans. In the present study, we have used a human physiologically-based pharmacokinetic (PBPK) model and an simple compartmental toxicokinetic model of benzo(a)pyrene (BaP) kinetics and its 3-hydroxybenzo(a)pyrene (3-OHBaP) metabolite to reproduce the time-course of this biomarker of exposure in the urine of industrially exposed workers and in turn predict the most plausible exposure scenarios. The models were constructed from *in vivo* experimental data in rats and then extrapolated from animals to humans after assessing and adjusting the most sensitive model parameters as well as species specific physiological parameters. Repeated urinary voids from workers exposed to polycyclic aromatic hydrocarbons (PAHs) have been collected over the course of a typical workweek and during subsequent days off work; urinary concentrations of 3-OHBaP were then determined. Based on the information obtained for each worker (BaP air concentration, daily shift hours, tasks, protective equipment), the time courses of 3-OHBaP in the urine of the different workers have been simulated using the PBPK and toxicokinetic models, considering the various possible exposure routes, oral, dermal and inhalation. Both models were equally able to closely reproduce the observed time course of 3-OHBaP in the urine of workers and predicted similar exposure scenarios. Simulations of various scenarios suggest that the workers under study were exposed mainly by the dermal route. Comparison of measured air concentration levels of BaP with simulated values needed to obtain a good approximation of observed time course further pointed out that inhalation was not the main route of exposure for most of the studied workers. Both kinetic models appear as a useful tool to interpret biomonitoring data of PAH exposure on the basis of 3-OHBaP levels.

## Introduction

Polycyclic aromatic hydrocarbons (PAHs) are a class of ubiquitous contaminants found in many industrial settings such as aluminum plants, silicon production plants and creosote impregnation plants [1], [2]. Workers are exposed through inhalation but also dermal contact depending on their job category, task and protective equipment [3]–[5]. Several members of this class of compounds have been categorized as probable or possible human carcinogens and the most studied PAH, benzo(a)pyrene (BaP), has been classified as a human carcinogen by the International Agency for Research on Cancer (IARC) [6].

Most industrial settings perform a strict monitoring of air levels of PAHs in their facilities, as well as skin patch analysis and/or in many cases rely on the biomonitoring of exposure of their staff [7]. Some years ago, Jongeneelen et al. [8], [9] proposed the use of 1-hydroxypyrene, a metabolite of the non-carcinogen PAH pyrene, as a biomarker of exposure to PAHs. More recently, 3-hydroxybenzo(a)pyrene (3-OHBaP) has been proposed as a complementary measure to better assess carcinogenic PAH exposure [7], [10]–[14]. Typical urinary levels of 3-OHBaP in workers have been reported to be around 0.5 nmol/mol creatinine while the general population values vary around 0.1 nmol/mol creatinine [10]. Campo et al. [15] assessed PAH exposure in coke-oven workers by determining urinary profiles of hydroxylated (including 3-OHBaP) and unmetabolized PAHs showing that both hydroxylated metabolites and unmetabolized PAHs in urine were useful biomarkers of exposure to PAHs. A few years ago, Forster et al. [10] assessed external and internal exposure to PAHs in 255 workers of different industries and the reliability of 3-OHBaP as a biomarker of internal exposure; BaP and 3-OHBaP were detected in the urine of workers from all workplaces. Also, positive correlations (r = 0.6 to 0.9) between urinary 3-OHBaP and 1-OHP as well as the sum of hydroxyphenols were found in workers of coke and graphite electrode production plants.

To help interpretation of biomonitoring data, toxicokinetic models have been proposed. Biologically-based toxicokinetic models allowing to relate the time course of a biomarker of exposure to the time-varying amounts in the body and doses per unit of time have been used by our group to reconstruct exposure in workers exposed to methanol and pesticides [16]–[19]. Recently, such type of toxicokinetic model has been developed to describe the kinetics of BaP and its 3-OHBaP biomarker of exposure in animals and humans [20]. Other types of kinetic models include physiologically-based pharmacokinetic (PBPK) models. Typically, these types of models are constructed from the interactions observed for a given substance with different organs, tissues and fluids *in vitro* [21]–[23], directly from the physicochemical characteristics of the substance under study [24] or simply by mathematically extrapolating its behaviour on the basis of similar compounds [25], [26]. An animal PBPK model of BaP and its 3-OHBaP metabolite was recently developed to describe the kinetics of BaP and 3-OHBaP [27]. The objective of our study was to extrapolate this PBPK model to humans and verify its use to reproduce time courses of 3-OHBaP in the urine of workers and predict the most plausible exposure scenarios. PBPK model predictions were also compared with those of a simple human toxicokinetic model based on rat data and relating urinary excretion of 3-OHBaP to whole body levels and BaP doses by different routes of exposure.

## Materials and Methods

### Establishment of a human PBPK model extrapolated from a rat PBPK model

#### Conceptual and functional representation

**Figure 1:**
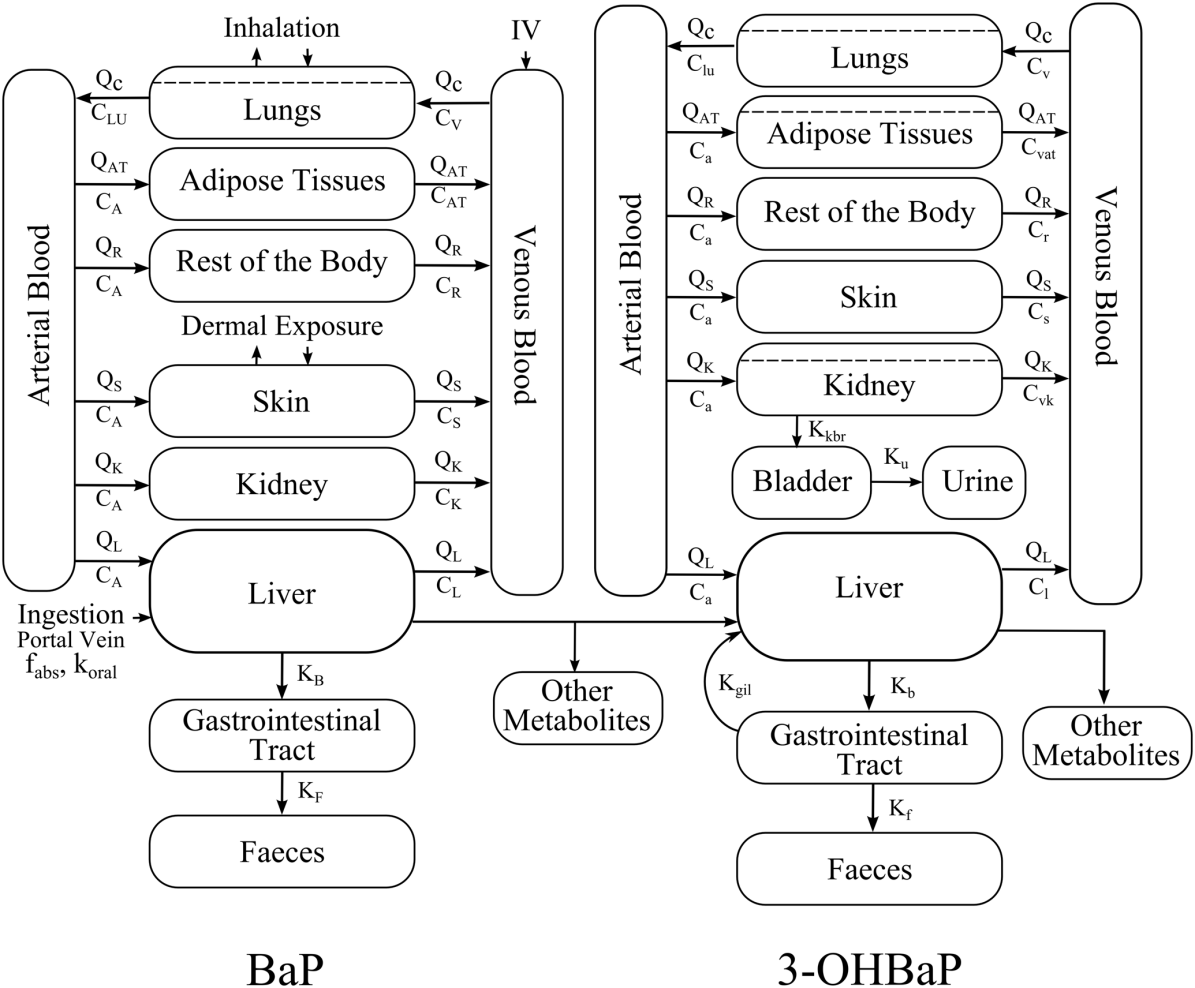
PBPK model of the kinetics of BaP and 3-OHBaP in humans.

A rat PBPK model representing the kinetics of BaP and its metabolite 3-OHBaP [27] was used as a basis for the establishment of a human-extrapolated model. The conceptual model is shown in Figure 1 and its mathematical representation is described in the Appendix S1. The kinetics of BaP and 3-OHBaP were simulated for three different routes of exposure: respiratory, dermal and oral exposures.

#### Human extrapolated model parameters

The rat physiological parameter values, such as organ weights, blood flow and total ventilation rates, were replaced by human values described in the medical literature by Davies and Morris [28] and Brown et al. [29]. Tissue: blood partition coefficients (describing perfusion-limited transfers) and tissue permeability coefficients (for diffusion-limited processes) were assumed to be species invariant; they were kept as established in the rat PBPK model, hence from *in vivo* time course data using a set of Monte Carlo algorithms with the Pearson χ^2^ as the best fit criteria [27], [30].

The other parameter values, which are known to be species specific, were best-fitted from available human data and include the metabolism rate, the excretion parameters and the dermal absorption parameters. The excretion parameters were adjusted from known rates (biliary excretion, renal filtration) in rats compared to humans [29] as described in Table 1. The metabolism rates of BaP and 3-OHBaP have been scaled from the rat model values as described in Table 1, and the BaP/3-OHBaP metabolism ratio was kept as obtained in rats. The rat-to-human scaling constant C_rat-human_ (defined as the ratio between human and rat metabolism rates) was computed directly from a least-square best-fit to an observed elimination time course of 3-OHBaP in the urine of a BaP exposed worker (using again the χ^2^ statistic as goodness of fit criteria). More specifically, the metabolism rate values of BaP and 3-OHBaP (taken to occur in the liver essentially) were determined by best-fit adjustments to the observed elimination time course of 3-OHBaP in urine over the 35 to 45-h period following the onset of a weekly exposure in worker 4, after setting human physiological parameter values and excretion rate values. The dermal absorption parameters were extrapolated as described in Table 1 on the basis of Morimoto’s scaling [31].

**Table 1:** Rat-to-human extrapolated key parameter values in the PBPK and toxicokinetic models^a^.

			PBPK model		Toxicokinetic model
Parameters	Units	Rats	Humans	Rats	Humans
Metabolic constants	1/h	BaP: V_max_/K_M_	BaP: C_rat-human_ bV_max_/K_M_		
	1/h	3-OHBaP: V_max_/K_M_	3-OHBaP: C_rat-human_ bV_max_/K_M_	k_b_	(V_AT_/Q_AT_)_rat_ (Q_AT_/V_AT_)_human_ (125/1.31) cC_rat-human_ bk_b_
Glomerular filtration rate	mL/h	K_kbr_	125/1.31 cK_kbr_		
Bileflow rate	mL/h	K_B_	350/22.5 dK_B_	-	-
Skin permeability coefficient	cm/h	k_P_	f_s_ ek_P_	D_dermal_(t)	f_s_ eD_dermal_(t)

a All the parameters are defined in the Appendix S1 with the usual nomenclature: V_max_ representing the maximum velocity rate of metabolism, K_m_ the Michaelis-Menten affinity rate constant, V_AT_ the adipose tissue volume and Q_AT_ the blood flow rate to adipose tissues.

b This is the scaling constant (C_rat-human_ = 1020.03) for rat-to-human extrapolation of metabolic constants.

c This is the ratio of glomerular filtration rates in rats (1.31 mL/min) and in humans (125 mL/min) as reported by Davies and Morris (1993).

d Values for the bile flow rate in rats (22.5 mL/day) and in humans (350 mL/day) from Davies and Morris (1993).

e According to the scaling proposed by Morimoto et al. (1992) relating the permeability coefficient in humans and hairless rat skin, and octanol-water partition coefficient, f_S_  =  (1.17×10^−7^ 6.19^0.751^+2.73×10^−8^)/(14.78×10^−7^ 6.19^0.589^+8.33×10^−8^).

#### Model simulation

Once all the parameters of the model have been fixed, simulations were carried out by numerically solving the system of differential equations representing the kinetics of BaP, on the one hand, and 3-OHBaP, on the other hand. All simulations were performed using Matlab 2010a (Mathworks, MA, USA).

#### Sensitivity simulation

Sensitivity analyses were performed to verify the stability of the overall model, by stochastically varying simultaneously all the parameter values of the model [32], [33]. This allowed testing the model for different routes of exposure and synergistic, combined and opposing effects of multiple parameter variations. We estimated the difference between the urinary excretion profiles resulting from all the parameter variations (1000 runs) and those obtained with the default parameter values (initially optimized), and calculated the mean percentages (± SD) of 20 data points (t = 8, 16, 24, 32, 40, 48, 56, 64, 72, 80, 88, 96, 104, 112, 120, 128, 136, 144, 152, and 160 h) falling within a 10% variation compared to initially optimized default parameter values. This allowed identifying the most sensitive parameters, as very sensitive parameters result in large variations in the urinary excretion profiles compared to less sensitive parameters, which cause very little urinary changes.

### Development of a simple compartmental toxicokinetic model

**Figure 2:**
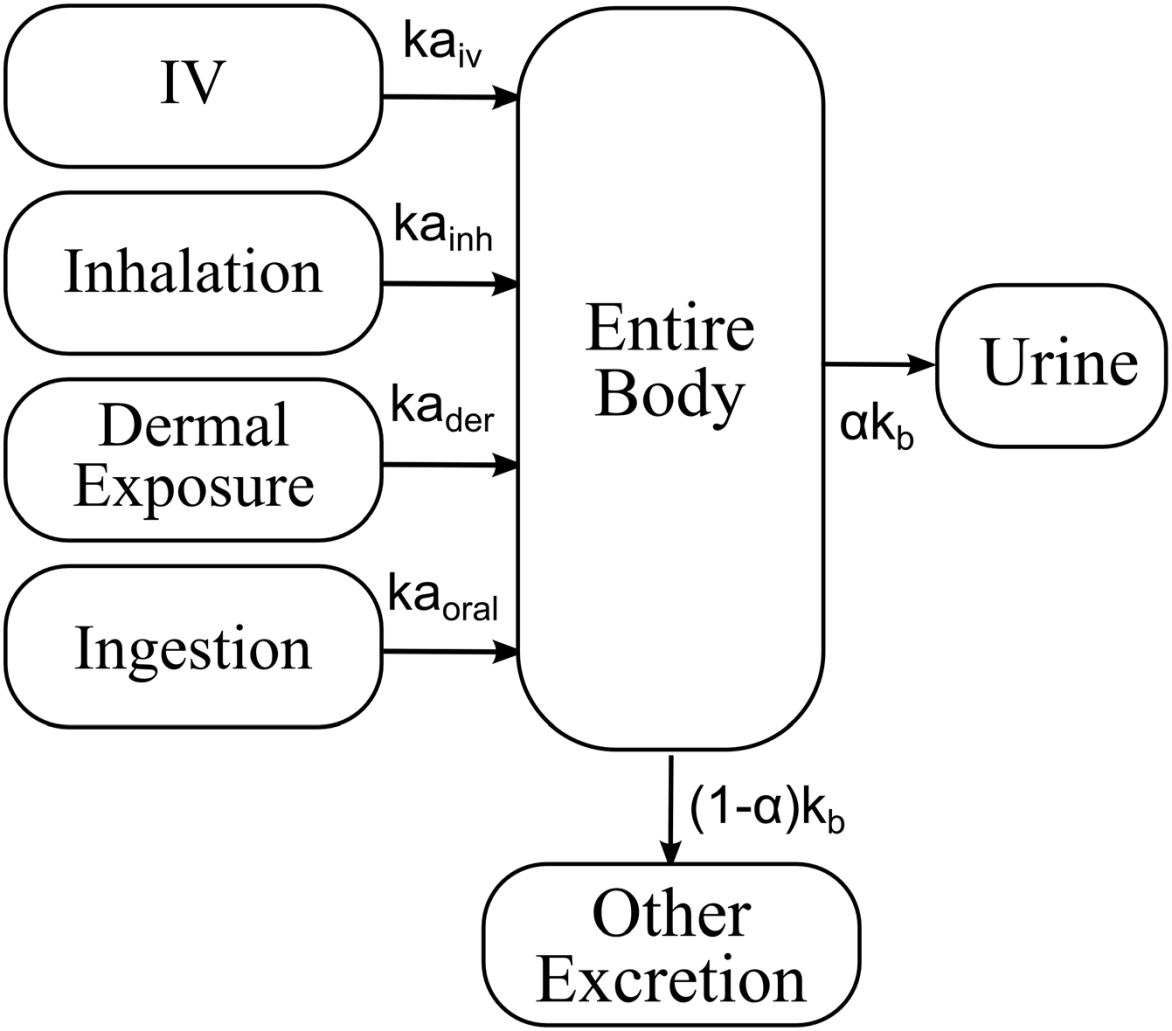
Single compartment model of the kinetics of BaP and 3-OHBaP in humans.

A simple one-compartment model representing BaP and 3-OHBaP in the whole body was built to relate urinary excretion time courses of 3-OHBaP with BaP absorbed doses by different routes-of-entry (Figure 2). The mathematical representation is described in the Appendix S1. The only key parameters in the model were the absorption rate (ka) of BaP by the different routes-of-entry (inhalation, ka_inh_; dermal, ka_der_; and oral, ka_oral_), the elimination rate from the body (k_b_) and the fraction of dose (α) recovered in urine as 3-OHBaP.

They were established on the basis of the same rat time course data [13], [34]–[36] used to build the rat PBPK model [27]. The Matlab 2010a built-in fit functions were used to determine rat parameter values, all at once, using the analytical solution of the differential equations. Key parameters in this model were the absorption rate by the different routes-of-exposure and the elimination rate from the body of BaP as 3-OHBaP, k_b_. Extrapolation of parameter values from rats to humans was heuristically determined as described in Table 1. As for the PBPK model, the dermal absorption parameter was extrapolated on the basis of Morimoto’s scaling [31] (Table 1). Extrapolation of the elimination rate from the body of BaP as 3-OHBaP, k_b_, accounted for species-specific changes in the metabolism rates and urinary elimination rate. This elimination rate is also influenced by the clearance of each organ. However, only the biological process contributing the most to the observed time-courses needed a species-specific adjustment, thus in our case the amounts of BaP in adipose tissues (according to Heredia-Ortiz et al. [20]) (see Table 1). Given the small number of parameters, no sensitivity analysis was carried out for the single compartment model. Once the parameters of the model were fixed, simulations of the same time courses as those reproduced with the human PBPK model were carried out in Matlab 2010a, using the exact solutions of the differential equations.

### PBPK and toxicokinetic model evaluation of 3-OHBaP time course data in workers

We assessed the capacity of our models to simulate urinary excretion time courses of 3-OHBaP in three groups of workers other than the one used to determine the human metabolic constants. Data provided for the time courses of 3-OHBaP in the urine of workers assessed by Lafontaine et al. and Gendre et al. [3], [5], [37] were simulated with the models. These included data from five subjects exposed in an artificial shooting target factory (including two Occupational Health practitioners on site for the biomonitoring and three full-time workers) and from five workers of a carbon disk brake production plant, respectively. A workshift personal air sampling of vapor and particulate PAHs was performed over two workdays for the assessment of atmospheric BaP concentrations, as described in Lafontaine et al. and Gendre et al. [3], [37]. Concentrations of 3-OHBaP were also determined as described in Simon et al. [38] in complete urine voids collected over the course of about a two-day work exposure and subsequent day with limited exposure. In the artificial shooting target factory, none of the workers wore respiratory protection but two of the workers wore gloves regularly. In the carbon disk brake production plant, one of the workers wore a cartridge mask and one wore a paper mask; the other workers did not wear respiratory protection equipment.

The third group monitored by the team of Professor Maître consisted of four employees repairing metallurgical furnaces in a silicon production plant. All workers assessed by the University Joseph-Fourier of Grenoble Hospital, France, gave their informed consent to participate in this study. In France, Occupational Health Physicians are mandated for the follow-up of workers, and perform routine biomonitoring of exposure. All samples were collected under the responsibility of the Occupational Health Physician of the company as part of the routine follow-up of workers and sent to the Grenoble Hospital. For laboratory analysis at the Hospital, personal identification coding were used and treated under the responsibility of a physician and a biologist sworn to medical secrecy. Results for each individual were then sent only to the Occupational Health Physician of the company, who interpreted the results to the workers. Only anonymous data was handed over to the researchers for this study.

Workers were aged between 28 and 54 years old and two of the four workers were smokers; they were assigned to the removal of plates and crowns surrounding the electrodes inside two ovens. This activity causes a significant release of PAHs in the air because electrodes are loaded with coal tar pitch. In addition, operators were in direct and indirect skin contact with the tar pitch. During this activity, no collective protection equipment was in place, but all the workers wore respiratory masks (with type ABEK2P2 or A2P3 cartridges) and leather handling gloves.

The disassembly of plates and crowns lasted four days (Tuesday to Friday), and employees were on a weekly rest during a two-day period before and after this task. A personal air sampling of vapor and particulate PAHs (n  = 19) was performed on the first and last day of the work week (Tuesday and Friday), in accordance to the French NF X 43–294 standard. Beginning and end-of-shift urine samples were also collected over the course of the work week. During the following 48-h period off work, workers were asked to collect all their micturitions in separate bottles. Polypropylene bottles were used for urine collection and samples were stored at −20°C until analysis. Concentrations of 3-OHBaP in urine were measured according to the method published by Barbeau et al. [39] and values were corrected for creatinine concentrations.

Model simulation of the observed urinary time courses of 3-OHBaP considering an inhalation and dermal exposure allowed assessing the influence of the route-of-exposure on the time profiles. Inhalation exposure scenarios, as assessed from measured BaP air concentrations and time-of-shifts during a workweek, served as an initial simulation of the time courses of 3-OHBaP in the urine of workers. Ventilation rate was taken to be 7.98 l/min for workers [28], [29]. Dermal exposure scenarios were simulated by considering a whole-body dermal dose of the same amounts of BaP diluted in about one liter. Changing BaP concentration or exposed surface only affects proportionally the simulated time-dependent concentration values, without affecting absorption or elimination slopes. Direct oral exposure was considered insignificant in those workers.

As a second independent step, in order to find the exposure doses that best described the urinary excretion profiles, a visual adjustment to the data points was first carried out. Then, a set of approximately a thousand doses around that value was established. A Monte Carlo simulation randomly picked values from this data set and checked for the corresponding χ^2^ statistic; at the end of a thousand iterations, the best value for each dose was kept.

## Results

### PBPK model extrapolated to humans and sensitivity analysis

The parameter values of the PBPK model are presented in Table 2. Using the model structure established from rat data (including entero-hepatic recirculation), the tissue-blood partition coefficients determined from the *in vivo* time-course data in rats, but a metabolism and elimination rate faster than in rats and slower dermal absorption rate, the PBPK model was able to simulate various sets of human time-course data.

**Table 2:** Human model parameters and sensitivity results.

			BaP		3-OHBaP	
	Matrix	Parameter	Value	Sensitivity^a^	Value	Sensitivity^a^
Partition coefficients	Lungs	P_LUA_ and P_lua_	2670.00	±39.6%	2.92	±39.6%
	Adipose tissues	P_ATV_ and P_atv_	65.90	±39.6%	1.42	±39.6%
	Skin	P_SV_ and P_sv_	1.87	±39.6%	0.80	±39.6%
	Kidneys	P_KV_ and P_kv_	2.08	±39.6%	40.40	±39.6%
	Liver	P_LV_ and P_lv_	12.90	±39.6%	1.83	±39.6%
	Rest of the body	P_RV_ and P_rv_	10.00	±29.6%	1.00	±39.6%
Permeability coefficients	Lungs	PA_LU_ and PA_lu_ [mL/h]	80.70	±39.6%	0.20	±39.6%
	Adipose tissues	PA_at_ [mL/h]	-	-	0.711	±39.6%
	Kidneys	PA_k_ [mL/h]	-	-	12.90	±39.6%
Metabolic Constants	Total metabolites	V_max_/K_M_ [mL/h]	951.69×10^3^	±38%	37.13×10^3^	±4.8%
	Fraction of 3-OHBaP	f_3OHBaP_ []	0.185	±4.0%	-	-
Elimination rates	Biliary	K_B_ and K_b_ [1/h]	0.338	±39.6%	663.80	±39.6%
	Urinary	K_kb_ [1/h]	-	-	60.40	±4.6%
		K_bu_ [1/h]	-	-	0.102	±12%
	Faecal	K_F_ and K_f_ [1/h]	0.334	±39.6%	0.173	±39.6%
		K_gil_ [1/h]	-	-	0.00693	±39.6%
Absorption constants	Dermal	k_P_ [cm/h]	0.00132	±39.6%	-	-
		P_DV_	1.0	±39.6%	-	-
	Inhalation	P_B_	2.04	±39.6%	-	-

a Range of parameter variation during Monte Carlo simulation to obtain 90.51 ± 1.15% of runs (n∼O(10^3^)) within a maximum variation of ±10% in the simulated urinary excretion profiles compared to default parameter values.

Monte Carlo simulations showed that the parameters influencing the most the overall excretion kinetics of 3-OHBaP were the metabolism rate and the elimination rate. Even a very small variation in values of the metabolic constants of BaP and 3-OHBaP can cause the simulation of the expected urinary excretion to change considerably (Table 2). In addition to the importance of the metabolic constants, we found that the elimination rates of 3-OHBaP through the kidneys and bladder largely determine the urinary excretion profiles. The fastest 3-OHBaP transfer rate is the transfer from the liver to the bile (K_b_ more than one orders of magnitude higher than the urinary elimination rate K_kb_), which leads directly to high levels of 3-OHBaP in faeces. The values of the other excretion parameters are similar, the K_bu_ value for the 3-OHBaP transferred from the bladder to urine being of the same order of magnitude as the fecal excretion K_F_ for BaP and K_f_ 3-OHBaP. These simulations also showed that the permeability coefficients are not the key parameters determining the overall time-course curves of BaP or 3-OHBaP in the various tissues. For instance, smaller permeability coefficients would primarily decrease concentrations at all times proportionally in organs limited by diffusion without significantly altering the curves of the other compartments.

### Single compartment toxicokinetic model

The toxicokinetic model parameter values for BaP absorption rate (ka) by inhalation (ka_inh_) and dermal contact (ka_der_) were respectively estimated to be: 48.35×10^−3 ^h^−1^ and 61.81×10^−3 ^h^−1^. The elimination rate from the body (k_b_) was equal to 198.26×10^−3 ^h^−1^, while the fractions of dose (α) recovered in urine as 3-OHBaP following inhalation and dermal exposure were found to be 3.99×10^−2^ and 1.64×10^−2^, respectively.

### PBPK and toxicokinetic model evaluation of 3-OHBaP time course data in workers

**Figure 3:**
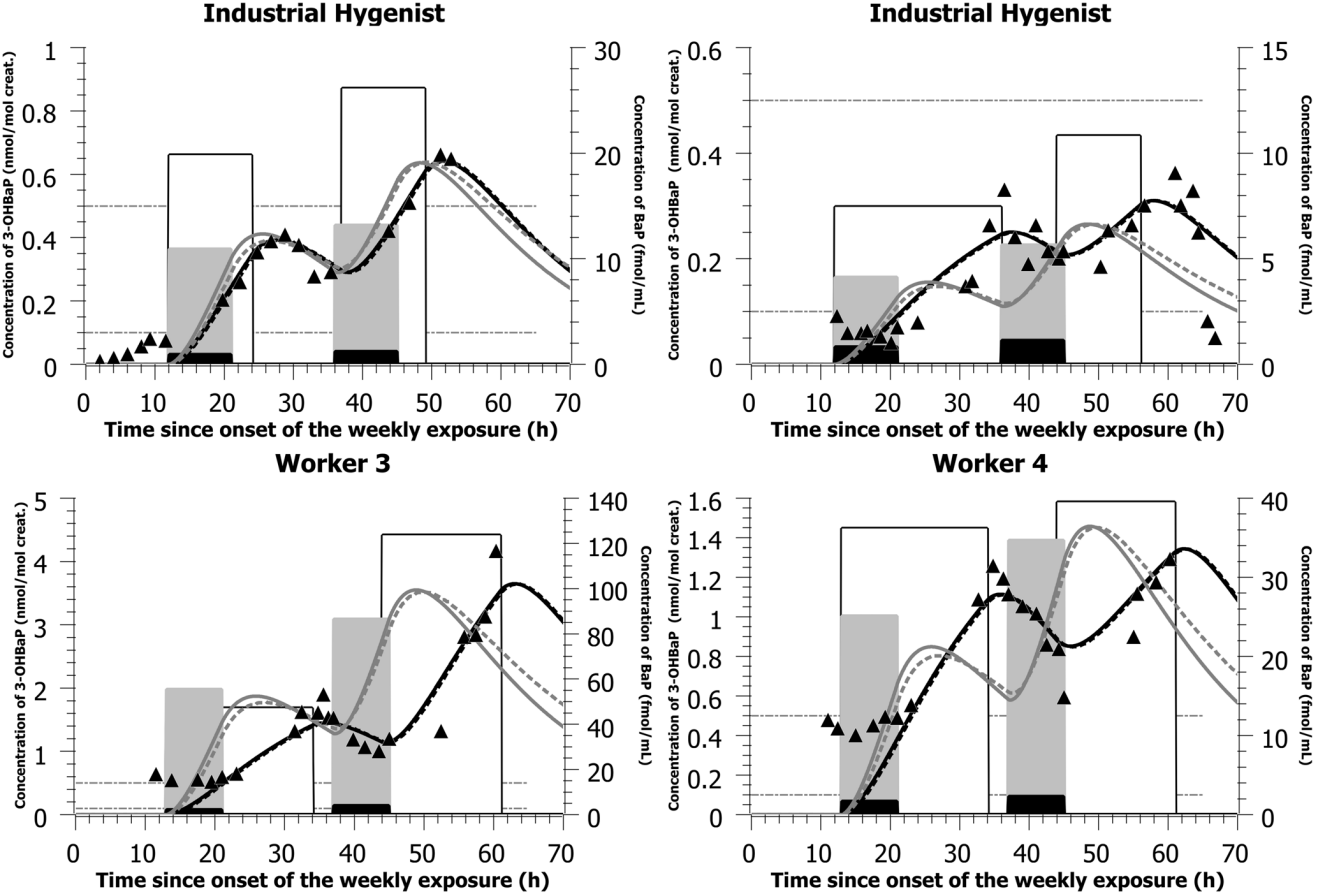
Model simulations of data from an artificial shooting target factory. Comparison of model simulations (lines) with observed data on the time courses of 3-OHBaP in the urine of subjects exposed to PAHs in an artificial shooting target factory (triangles - left-axis). The light gray bars (right-axis) indicate the simulated BaP inhalation exposure scenarios (concentration and time), the white bars (right-axis) indicate the simulated BaP dermal exposure scenarios (concentration and time) while the black bars (right-axis) show the measured inhalation exposure scenarios (measured air concentration (ng/m^3^ converted to fmol/mL) and documented time-of-shift; see also Table 3). The black solid lines represent PBPK model simulation considering an exposure by the dermal route solely while the dark gray solid lines represent a simulated inhalation. The black dotted lines represent toxicokinetic model simulation considering a dermal exposure solely while the dark gray dotted lines represent a simulated exposure by inhalation. All inhalation concentrations measured in ng/m^3^ were expressed in nmol/m^3^ and converted to fmol/mL (multiplied by 10^3^ so that all the scenarios could be graphically represented on the same figure for comparison).

**Figure 4:**
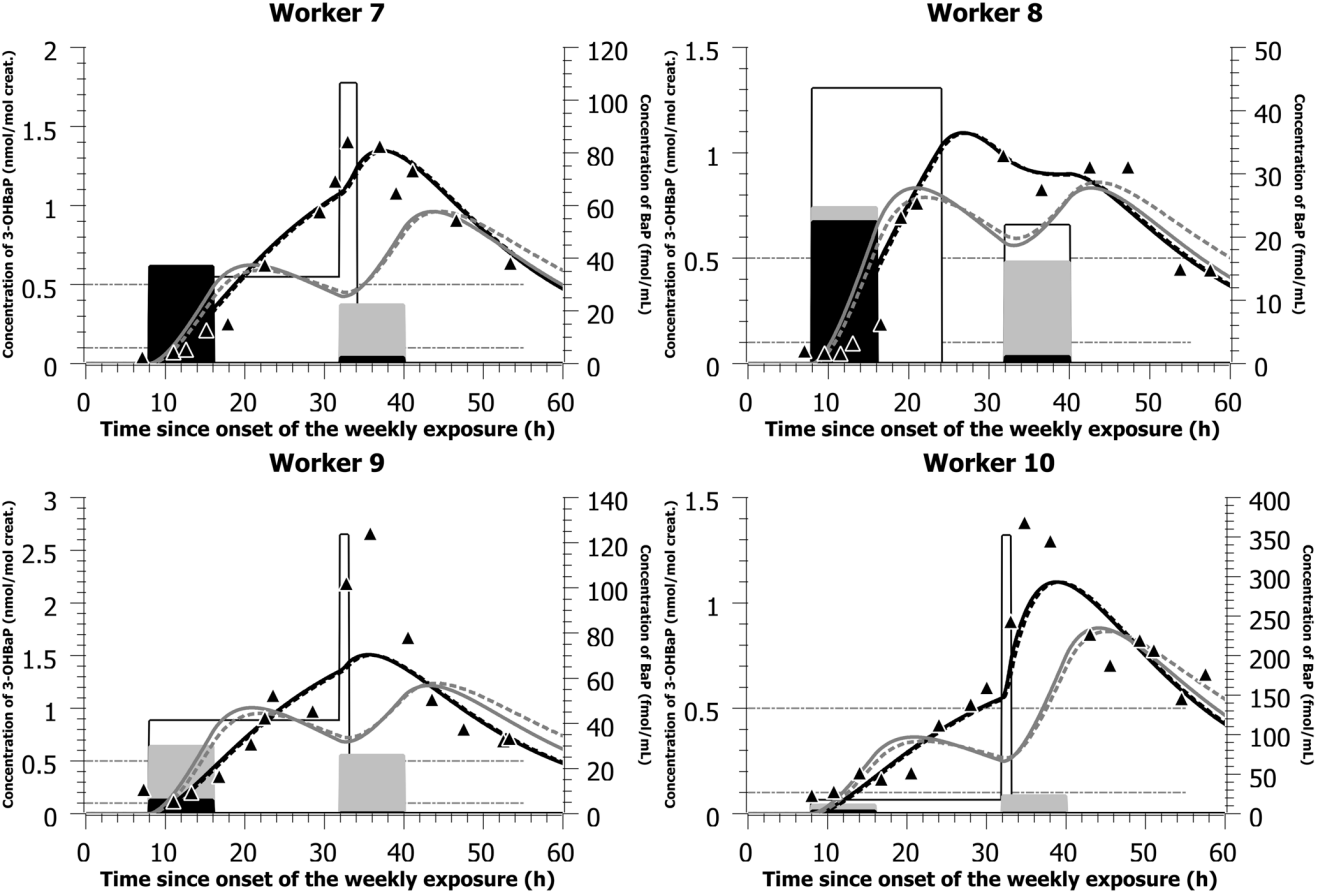
Model simulations of data from a carbon disk brake production factory. Comparison of model simulations (lines) with observed data on the time courses of 3-OHBaP in the urine of workers exposed to PAHs in a carbon disk brake production plant (triangles - left-axis). The light gray bars (right-axis) indicate the simulated BaP inhalation exposure scenarios (concentration and time), the white bars (right-axis) indicate the simulated BaP dermal exposure scenarios (concentration and time) while the black bars (right-axis) show the measured inhalation exposure scenarios (measured air concentration (ng/m^3^ converted to fmol/mL) and documented time-of-shift; see also Table 4). The black solid lines represent PBPK model simulation considering an exposure by the dermal route solely while the dark gray solid lines represent a simulated inhalation. The black dotted lines represent toxicokinetic model simulation considering a dermal exposure solely while the dark gray dotted lines represent a simulated exposure by inhalation. All inhalation concentrations measured in ng/m^3^ were expressed in nmol/m^3^ and converted to fmol/mL (multiplied by 10^3^ so that all the scenarios could be graphically represented on the same figure for comparison).

**Figure 5:**
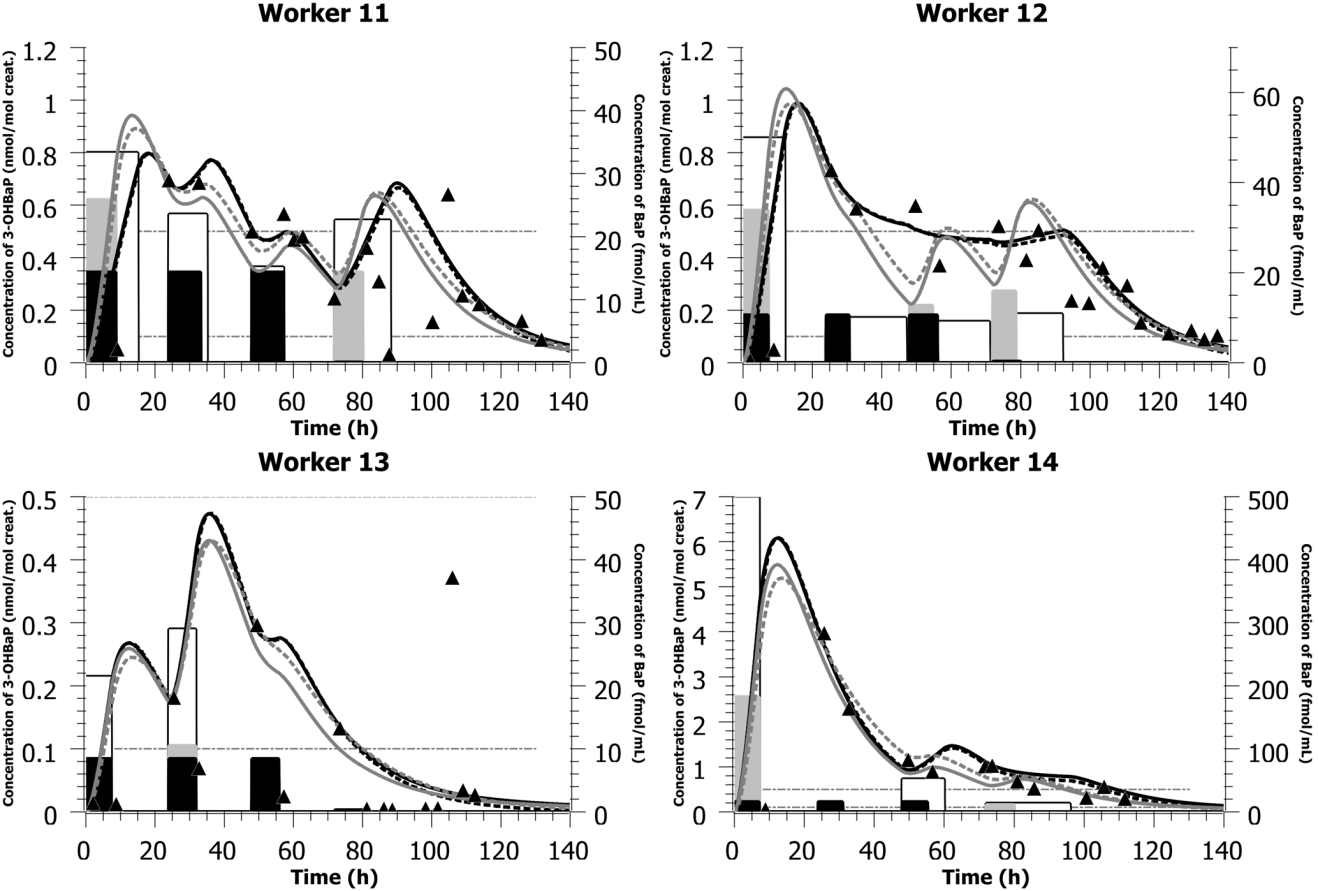
Model simulations of data from a silicon production factory. Comparison of model simulations (lines) with observed data on the time courses of 3-OHBaP in the urine of workers exposed to PAHs in a silicon production industry (triangles - left-axis). The light gray bars (right-axis) indicate the simulated BaP inhalation exposure scenarios (concentration and time), the white bars (right-axis) indicate the simulated BaP dermal exposure scenarios (concentration and time) while the black bars (right-axis) show the measured inhalation exposure scenarios on days 1 and 4, with values on days 2 and 3 considered similar to day 1 (measured air concentration (ng/m^3^ converted to fmol/mL) and documented time-of-shift; see also Table 5). The black solid lines represent PBPK model simulation considering an exposure by the dermal route solely while the dark gray solid lines represent a simulated inhalation. The black dotted lines represent toxicokinetic model simulation considering a dermal exposure solely while the dark gray dotted lines represent a simulated exposure by inhalation. All inhalation concentrations measured in ng/m^3^ were expressed in nmol/m^3^ and converted to fmol/mL (multiplied by 10^3^ so that all the scenarios could be graphically represented on the same figure for comparison).

With the parameter values presented in Table 2 and the simulated mostly dermal exposure scenarios (concentrations and time-of-shift) presented in Tables 3 and 4, the PBPK model was able to reproduce closely the time courses of 3-OHBaP in the urine of individuals exposed to PAHs in two different plants, with complete time-voids over the course of a workweek (Figures 3 and 4). The model was also able to reproduce the times profiles observed in a third industry where workers provided spot samples prior, during and following a work period (Figure 5 and Table 5). PBPK model simulations were compared to predictions obtained with a simple compartmental toxicokinetic model and Figures 3, 4, 5 show that both models provided similar fits to the observed data. For most workers, a more pronounced difference was observed between simulated and observed time courses when considering an exposure by inhalation. Therefore, we have optimized for dermal exposure scenarios, which considered for most workers an exposure not only during their work-shift hours but also a continued absorption after work task hours. Simulations thus suggest a dermal contamination of BaP during work, without a proper cleaning thereafter.

**Table 3:** Simulated dermal and inhalation exposure scenarios compared with measured BaP inhalation exposure scenario (air concentrations and time-of-shifts) in subjects exposed to PAHs in an artificial shooting target factory (observed data from [37]).

Subject^a^	Route-of-entry	Measured exposure scenarios		Simulated exposure scenarios	
		Day 1	Day 2	Day 1	Day 2
1	Inhalation	233 ng/m^3^12∶00–21∶00	302 ng/m^3^12∶00–21∶00	2749.4 ng/m^3^12∶00–21∶00	3322 ng/m^3^12∶00–21∶00
	Dermal exposure			19.8 fmol/mL12∶00–24∶00	26.1 fmol/mL13∶00–1∶00
2	Inhalation	208 ng/m^3^12∶00–21∶00	285 ng/m^3^12∶00–21∶00	1040 ng/m^3^12∶00–21∶00	1425 ng/m^3^12∶00–21∶00
	Dermal exposure			7.5 fmol/mL12∶00–12∶00	10.8 fmol/mL8∶00–20∶00
3	Inhalation	537 ng/m^3^13∶00–21∶00	991 ng/m^3^13∶00–21∶00	13962 ng/m^3^13∶00–21∶00	21802 ng/m^3^13∶00–21∶00
	Dermal exposure			47.1 fmol/mL13∶00–10∶00	123.6 fmol/mL20∶00–13∶00
4^b^	Inhalation	422 ng/m^3^13∶00–21∶00	583 ng/m^3^13∶00–21∶00	6330 ng/m^3^13∶00–21∶00	8745 ng/m^3^13∶00–21∶00
	Dermal exposure			36.2 fmol/mL13∶00–10∶00	39.5 fmol/mL20∶00–13∶00
5^c^	Inhalation	519 ng/m^3^13∶00–21∶00	684 ng/m^3^13∶00–21∶00	7785 ng/m^3^13∶00–21∶00	8208 ng/m^3^13∶00–21∶00
	Dermal exposure			36.2 fmol/mL13∶00–10∶00	56.8 fmol/mL20∶00–16∶00

a Subjects 1 and 2 were the industrial hygienists on site for the biomonitoring of exposure to PAH in workers. None of the workers wore respiratory protection but two of the workers wore gloves regularly.

b This worker was used to fit the human metabolism rate of BaP and 3-OHBaP.

c Worker 5 not presented in Figure 3.

**Table 4:** Simulated dermal and inhalation exposure scenarios compared with measured BaP inhalation exposure scenario (air concentrations and time-of-shifts) in workers exposed to PAHs in a carbon disk brake production plant (observed data from [3]).

Worker^a^	Route-of-entry	Measured exposure scenarios		Simulated exposure scenarios	
		Day 1	Day 2	Day 1	Day 2
6^b^	Inhalation	8 ng/m^3^8∶00–16∶00		240 ng/m^3^8∶00–16∶00	240 ng/m^3^8∶00–16∶00
	Dermal exposure			2 fmol/mL8∶00–16∶00	4.6 fmol/mL8∶00–16∶00
7	Inhalation	9300 ng/m^3^8∶00–16∶00	560 ng/m^3^8∶00–16∶00	4650 ng/m^3^8∶00–16∶00	5600 ng/m^3^8∶00–16∶00
	Dermal exposure			32.6 fmol/mL8∶00–8∶00	106.3 fmol/mL8∶00–10∶00
8	Inhalation	5650 ng/m^3^8∶00–16∶00	270 ng/m^3^8∶00–16∶00	6215 ng/m^3^8∶00–16∶00	4050 ng/m^3^8∶00–16∶00
	Dermal exposure			43.5 fmol/mL8∶00–24∶00	21.9 fmol/mL8∶00–16∶00
9	Inhalation	1500 ng/m^3^8∶00–16∶00	65 ng/m^3^8∶00–16∶00	7500 ng/m^3^8∶00–16∶00	6500 ng/m^3^8∶00–16∶00
	Dermal exposure			41 fmol/mL8∶00–8∶00	123.4 fmol/mL8∶00–9∶00
10	Inhalation	775 ng/m^3^8∶00–16∶00	63 ng/m^3^8∶00–16∶00	2712.5 ng/m^3^8∶00–16∶00	5670 ng/m^3^8∶00–16∶00
	Dermal exposure			16.6 fmol/mL8∶00–8∶00	351.6 fmol/mL8∶00–9∶00

a Worker 7 wore a paper mask; worker 6, 7 and 9 did not wear any respiratory protection equipment; worker 10 wore a cartridge mask.

b Worker 6 not presented in Figure 4.

**Table 5:** Simulated dermal and inhalation exposure scenarios compared with measured BaP inhalation exposure scenario (air concentrations and time-of-shifts) in workers exposed to PAHs during a metallurgical furnace repair in a silicon production plant.

Worker	Units	Measured exposure scenarios				Simulated exposure scenarios			
		Day 1	Day 2	Day 3	Day 4	Day 1	Day 2	Day 3	Day 4
11^a^	Inhalation	3619 ng/m^3b^6∶00–14∶00	ND^b^^,^ c	ND^b^^,^ c	18.5 ng/m^3^6∶00–14∶00	6514.2 ng/m^3^6∶00–14∶00	1881.8 ng/m^3^6∶00–14∶00	1737.1 ng/m^3^6∶00–14∶00	3619 ng/m^3^6∶00–14∶00
	Dermal exposure					33.3 fmol/mL6∶00–21∶00	23.6 fmol/mL6∶00–17∶00	15.1 fmol/mL6∶00–14∶00	22.6 fmol/mL6∶00–22∶00
12^a^	Inhalation	2668.1 ng/m^3b^6∶00–14∶00	ND^b^^,^ c	ND^b^^,^ c	77 ng/m^3^6∶00–14∶00	8537.9 ng/m^3^6∶00–14∶00	800.4 ng/m^3^6∶00–14∶00	3201.7 ng/m^3^6∶00–14∶00	4002.1 ng/m^3^6∶00–14∶00
	Dermal exposure					49.9 fmol/mL6∶00–18∶00	10 fmol/mL6∶00–5∶00	9.1 fmol/mL6∶00–5∶00	10.8 fmol/mL6∶00–2∶00
13^a^	Inhalation	2121.5 ng/m^3b^6∶00–14∶00	ND^b^^,^ c	ND^b^^,^ c	28 ng/m^3^6∶00–14∶00	2121.5 ng/m^3^6∶00–14∶00	2651.9 ng/m^3^6∶00–14∶00	424.3 ng/m^3^6∶00–14∶00	28 ng/m^3^6∶00–14∶00
	Dermal exposure					21.5 fmol/mL6∶00–14∶00	29 fmol/mL6∶00–14∶00	7.8 fmol/mL6∶00–14∶00	0.3 fmol/mL6∶00–14∶00
14^a^	Inhalation	3996.7 ng/m^3b^6∶00–14∶00	ND^b^^,^ c	ND^b^^,^ c	51 ng/m^3^6∶00–14∶00	45962 ng/m^3^6∶00–14∶00	399.7 ng/m^3^6∶00–14∶00	3996.7 ng/m^3^6∶00–14∶00	2805 ng/m^3^6∶00–14∶00
	Dermal exposure					498.6 fmol/mL6∶00–14∶00	0 fmol/mL6∶00–14∶00	52 fmol/mL6∶00–18∶00	13.3 fmol/mL6∶00–6∶00

a Workers were exposed during 4 consecutive days (Tuesday to Friday). No collective protection equipment was in place, but workers wore masks (with type ABEK2P2 or A2P3 cartridges) and leather handling gloves.

b Days 1 to 4 correspond to the four exposure days, hence days where workers were performing repair tasks exposing them to PAHs. However, air concentrations were measured on the first exposure day of the week (Tuesday) by the team of Professor Maître and taken to be equal on days 2 and 3 of exposure (Wednesday, Thursday). Air concentrations were also monitored on the last exposure day (Friday; day 4 of exposure).

c Not determined.

Furthermore, the inhaled dose scenarios simulated to reproduce the urinary profiles were in most cases higher than those recorded from airborne measurements of BaP concentrations in the facilities and time-of-shifts (Tables 3, 4, 5). Cases in which the simulated inhaled BaP concentration levels needed to obtain a better fits to observed profiles were much higher than measured air concentration values further indicate that inhalation was not the main route of exposure. Therefore, it suggests a mostly dermal exposure for workers of the artificial shooting target factory (workers 1 to 5) and the carbon disk brake production plant (workers 6 to 10, except maybe worker 8 on day one where simulating an inhalation exposure resulted in good predictions) as well as worker 12 of the silicon production industry, evidently, but also possibly some of the other workers (workers 11 and 14). In addition, for workers of the silicone production plant performing oven repairs (Figure 5 and Table 5), we have considered in the initial modeling that BaP air concentrations on the first three exposure days (Tuesday to Thursday) were the same as those measured on the first exposure day of the week (Tuesday, where atmospheric measurements were available). However, there might be significant variations in BaP concentrations throughout the week as values measured on day four of exposure (Friday) were very different from those measured on the first day of the week (two orders of magnitude).

Interestingly, Figure 3 allows comparing 3-OHBaP excretion profiles in Occupational Health practitioners on site to perform the biomonitoring of workers (physician as subject 1 and nurse as subject 2) with those of industry workers. In line with observed time course data, low exposures were simulated at the onset of collection period with a progressive rise above typical general population values by the end of the first exposure day and values reaching those observed in workers by the end of the second day of exposure. On the other hand, the observed time profiles in workers (subjects 3–5) show a background exposure at the onset of workweek (with corresponding urinary values around 0.5 nmol/mol creat.) with a progressive rise over the course of workweek. In workers of the second industry, a similar pattern was observed (Figure 4).

Figure 5 also shows a good adequacy between model simulations and observed data in workers of the third industry. Two of the workers (11 and 12) exhibited similar profiles, with modeled high exposure at the beginning of the workweek and ensuing decreasing exposures throughout the week (with daily peaks and troughs). The most highly exposed worker was also modeled with a clear exposure mainly on day 1 and progressive decrease thereafter (worker 14) whereas worker 13 showing the lowest exposure to BaP exhibited the most erratic profiles (and hence excretion values <0.4 nmol/mol creat.).

## Discussion

Current risk assessment studies attempt to establish reliable tools enabling to reconstruct absorbed doses or exposure doses in individuals from measurements of exposure biomarkers in accessible biological matrices. In our study, we have ascertained a one-to-one relation between the studied exposure dose and 3-OHBaP biomarker in urine using a thorough knowledge of the toxicokinetics of BaP and 3-OHBaP encoded in two mathematical models, which describe the major determinants of the excretion kinetics.

The development of two types of toxicokinetic models in humans, based on *in vivo* time course data of BaP and 3-OHBaP in rats, as obtained from the available literature, proved useful to reproduce adequately the excretion time courses of 3-OHBaP biomarker in workers and predict the most plausible exposure scenarios. Although the mathematical representations of the two types of models differ, parameter values of both models were easily extrapolated from animals to humans using extrapolation factors and in turn predicted similar exposures. However, the PBPK model, with a more physiological description of the internal kinetics and based on a rat model evaluated with detailed time course data on the internal kinetics in animals [27], allowed pointing out the critical determinants of the overall excretion kinetics. The rat PBPK model indicated that the metabolic rates of both BaP and 3-OHBaP (with a major contribution of the metabolism from the liver over the lungs and negligible relative skin metabolism) and excretion rate of 3-OHBaP were the most sensitive parameters governing the observed 3-OHBaP urinary excretion kinetics. The rat PBPK model also showed that the skin permeability coefficient and tissue-permeability coefficients for diffusion-limited processes were not the key parameters determining the overall time course curves of BaP or 3-OHBaP in the various tissues; a smaller skin permeability coefficient, as expected in humans compared to rats, would only decrease concentrations at all times proportionally in organs without significantly altering the shape of the curves. The PBPK modeling of rat data [27] further showed that the slow release of BaP from adipose tissues and lungs (possibly lipid components of the lung) was the rate-limiting step driving the overall observed time profiles of BaP in blood, even following a dermal exposure where absorption rate is expected to be slower than following inhalation exposure. By setting these key parameter values of the PBPK model to human specific values, the rat-based PBPK model provided very good fits to the available worker time course data.

Both the PBPK and toxicokinetic modeling of BaP and 3-OHBaP have also shown that inference of the main route-of-entry on the basis of model fitting of observed excretion time courses of 3-OHBaP, considering an inhalation or dermal exposure, is not straightforward. The available number of sampling points to describe the excretion profiles of 3-OHBaP could be reproduced by several plausible exposure scenarios. Therefore, unless one has information on worker tasks or exposure concentrations, the resulting urinary biomarker profiles alone do not allow confirming the main route of BaP exposure. Our model has also shown that, a few hours after exposure, the urinary excretion profiles are governed by the slow release of BaP from organs retaining the parent compound, namely adipose tissues and lungs. A similar observation can be drawn from a toxicokinetic model previously developed by our team [20]. Also, according to this PBPK modeling and prior toxicokinetic modeling [20] based on observed time-course data in rats [35], [40], the urinary excretion of 3-OHBaP is delayed with respect to blood profiles. Our modeling assumed that the major reason for such a delay was the relatively slow transfer of 3-OHBaP occurring in the kidneys and a possible interaction of 3-OHBaP in the bladder.

Overall, our kinetic modeling provided a tool to better interpret 3-OHBaP biomonitoring data in workers exposed to PAHs. It pointed out that there is a need for sufficient time course points in workers over a workweek (and ideally, complete urine voids) to be able to reconstruct exposure. In the modeling process, it remains important to consider information on tasks performed by workers and air concentrations, as was done by other authors [3], [5], [10], [37], to better assess potential major route-of-exposure. The use of multiple biomarker measurements, such a 1-hydroxypyrene (1-OHP) and 3-OHBaP in combination, may further help interpreting biomonitoring results, especially given that BaP is a substance that is more representative of carcinogenic PAHs than pyrene, as highlighted by others [10]. In addition, there are differences in the excretion kinetics of 1-OHP and 3-OHBaP [3], [40], such that 1-OHP excretion profile is more obviously influenced by the main route-of-entry (inhalation versus dermal) compared with 3-OHBaP. This has been observed in a recent study, in which the urinary excretions of 1-OHP in humans exposed to PAHs have been compared to simulations obtained with a PBPK model for pyrene [41]. In the latter study, simulated urinary profiles presented different time-to-peak levels for each route of exposure: less than 8 h for inhalation in electrode paste workers and around 15 h for volunteers dermally exposed. The PBPK model developed for pyrene [41] differs from current BaP and 3-OHBaP model in the sense that essential parameters such as the tissue-blood partition coefficients were established from *in vivo* time courses rather than *in vitro*; specific determinants of BaP and 3-OHBaP kinetics were also accounted for, such as chemical-specific metabolism and atypical renal excretion, and differences in elimination rates and entero-hepatic recirculation.

## Supporting Information

Appendix S1 **Kinetic equations.**
(DOCX)
