# Supplementary material for: Use of Physiologically-Based Pharmacokinetic Modeling to Simulate the Profiles of 3-Hydroxybenzo(a)pyrene in Workers Exposed to Polycyclic Aromatic Hydrocarbons
Source: PLoS One. 2014 Jul 17;9(7):e102570. doi: 10.1371/journal.pone.0102570 (PMC4102510; doi:10.1371/journal.pone.0102570)
Supplement: Appendix S1 — Kinetic equations. (DOCX) [file pone.0102570.s001.docx]

# Appendix S1: Kinetic equations

Differential equations defining both models are described. First, the mathematical representation of the PBPK model is presented. As usual, C stands for concentrations (μmol/mL), A for amounts (μmol), Q for regional blood flow rate, V for volumes, P for tissue:blood partition coefficients, PA for permeability-area coefficients, f for fractions (e.g. fraction of blood in tissues), k_P_ for skin permeability coefficient, K for rates, S surface of exposure, V_MAX_ total maximum rate of metabolism and K_M_ Michaelis constant. Tissues and fluids are labeled in indices as follows for BaP: LU for lungs, AT for adipose tissues, V for venous blood, A for arterial blood, S for skin, K for kidneys, L for liver, R for the rest of the body, GI for gastrointestinal tract, F for faeces and U for urine. Tissues and fluids are labeled as follows for 3-OHBaP: lu for lungs, at for adipose tissues, v for venous blood, a for arterial blood, s for skin, k for kidneys, l for liver, r for the rest of the body, gi for gastrointestinal tract, br for bladder, f for faeces and u for urine. Second, the mathematical representation of the toxicokinetic model is presented. D is the dose absorbed, k_a_ are the rates of absorption, k_b_ is the rate of elimination and α is the fraction of 3-OHBaP eliminated through urine.

**PBPK modeling of BaP kinetics**

**Kinetics of BaP**

Lungs

$f_{LU}V_{LU}\frac{\partial}{\partial t}C_{VLU}(t)=Q_{C}C_{V}(t)+Q_{P}C_{I}(t)-\left(Q_{C}+\frac{Q_{P}}{P_{B}}\right)C_{VLU}(t)-PA_{LU}\left[C_{VLU}(t)-\frac{C_{LU}(t)}{P_{LUA}}\right]$ , (1)

$\left(1-f_{LU}\right)V_{LU}\frac{\partial}{\partial t}C_{LU}(t)=PA_{LU}\left[C_{VLU}(t)-\frac{C_{LU}(t)}{P_{LUA}}\right]$, (2)

Adipose tissues

$V_{AT}\frac{\partial}{\partial t}C_{AT}(t)=Q_{AT}C_{A}(t)-\frac{Q_{AT}}{P_{ATV}}C_{AT}(t)$ , (3)

Skin

$V_{S}\frac{\partial}{\partial t}C_{S}(t)=Q_{S}C_{A}(t)-\frac{Q_{S}}{P_{SV}}C_{S}(t)+k_{P}S\left[C_{D}(t)-\frac{C_{S}(t)}{P_{DV}}\right]$, (4)

Kidneys

$V_{K}\frac{\partial}{\partial t}C_{K}(t)=Q_{K}C_{A}(t)-\frac{Q_{K}}{P_{KV}}C_{K}(t)$, (5)

Liver

$V_{L}\frac{\partial}{\partial t}C_{L}(t)=Q_{L}C_{A}(t)-\left(\frac{Q_{L}}{P_{LV}}+\frac{V_{MAX}}{K_{M}P_{LV}}+K_{B}\right)C_{L}(t)+f_{abs}^{ORAL}K_{ORAL}A_{ORAL}(t)$, (6)

Blood

$f_{AB}V_{B}\frac{\partial}{\partial t}C_{A}(t)=Q_{C}C_{VLU}(t)-Q_{C}C_{A}(t)$, (7)

$$\left(1-f_{AB}\right)V_{B}\frac{\partial}{\partial t}C_{V}(t)=C_{IV}(t)-Q_{C}C_{V}(t)+\frac{Q_{AT}}{P_{ATV}}C_{AT}(t)+\frac{Q_{S}}{P_{SV}}C_{S}(t)+\frac{Q_{K}}{P_{KV}}C_{K}(t)+\frac{Q_{L}}{P_{LV}}C_{L}(t)+\frac{Q_{R}}{P_{RV}}C_{R}(t)$$

, (8)

Rest of the body

$V_{R}\frac{\partial}{\partial t}C_{R}(t)=Q_{R}C_{A}(t)-\frac{Q_{R}}{P_{RV}}C_{R}(t)$, (9)

Gastrointestinal tract

$\frac{\partial}{\partial t}A_{GI}(t)=K_{B}C_{L}(t)-K_{F}A_{GI}(t)$, (10)

Faeces

$\frac{\partial}{\partial t}A_{F}(t)=K_{F}A_{GI}(t)$. (11)

**Kinetics of 3-OHBaP**

Lungs

$f_{LU}V_{LU}\frac{\partial}{\partial t}C_{vlu}(t)=Q_{C}C_{v}(t)+Q_{C}C_{vlu}(t)-PA_{lu}\left[C_{vlu}(t)-\frac{C_{lu}(t)}{P_{lua}}\right]$, (12)

$\left(1-f_{LU}\right)V_{LU}\frac{\partial}{\partial t}C_{lu}(t)=PA_{lu}\left[C_{vlu}(t)-\frac{C_{lu}(t)}{P_{lua}}\right]$, (13)

Adipose tissues

$f_{AT}V_{AT}\frac{\partial}{\partial t}C_{vat}(t)=Q_{AT}C_{a}(t)+Q_{AT}C_{vat}(t)-PA_{at}\left[C_{vat}(t)-\frac{C_{at}(t)}{P_{atv}}\right]$, (14)

$\left(1-f_{AT}\right)V_{AT}\frac{\partial}{\partial t}C_{at}(t)=PA_{at}\left[C_{vat}(t)-\frac{C_{at}(t)}{P_{atv}}\right]$, (15)

Skin

$V_{S}\frac{\partial}{\partial t}C_{s}(t)=Q_{S}C_{a}(t)-\frac{Q_{S}}{P_{sv}}C_{s}(t)$, (16)

Kidneys

$f_{K}V_{K}\frac{\partial}{\partial t}C_{vk}(t)=Q_{K}C_{a}(t)+\left(Q_{K}+K_{kbr}\right)C_{vk}(t)-PA_{k}\left[C_{vk}(t)-\frac{C_{k}(t)}{P_{kv}}\right]$, (17)

$\left(1-f_{K}\right)V_{K}\frac{\partial}{\partial t}C_{k}(t)=PA_{k}\left[C_{vk}(t)-\frac{C_{k}(t)}{P_{kv}}\right]$, (18)

Liver

$V_{L}\frac{\partial}{\partial t}C_{l}(t)=Q_{L}C_{a}(t)-\left(\frac{Q_{L}}{P_{lv}}+\frac{V_{\max}}{K_{m}P_{lv}}+K_{b}\right)C_{l}(t)+K_{gil}A_{gi}(t)+\frac{V_{MAX}}{K_{M}P_{LV}}C_{L}(t)$, (19)

Blood

$f_{AB}V_{B}\frac{\partial}{\partial t}C_{a}(t)=Q_{C}C_{vlu}(t)-Q_{C}C_{a}(t)$, (20)

$\left(1-f_{AB}\right)V_{B}\frac{\partial}{\partial t}C_{v}(t)=-Q_{C}C_{v}(t)+Q_{AT}C_{vat}(t)+\frac{Q_{S}}{P_{sv}}C_{s}(t)+Q_{K}C_{vk}(t)+\frac{Q_{L}}{P_{lv}}C_{l}(t)+\frac{Q_{R}}{P_{rv}}C_{r}(t)$, (21)

Rest of the body

$V_{R}\frac{\partial}{\partial t}C_{r}(t)=Q_{R}C_{a}(t)-\frac{Q_{R}}{P_{rv}}C_{r}(t)$, (22)

Gastrointestinal tract

$\frac{\partial}{\partial t}A_{gi}(t)=K_{b}C_{l}(t)-\left(K_{f}+K_{gil}\right)A_{gi}(t)$, (23)

Faeces

$\frac{\partial}{\partial t}A_{f}(t)=K_{f}A_{gi}(t)$, (24)

Bladder

$\frac{\partial}{\partial t}A_{br}(t)=K_{kbr}C_{vk}(t)-K_{u}A_{br}(t)$, (25)

Urine

$\frac{\partial}{\partial t}A_{u}(t)=K_{u}A_{br}(t)$. (26)

**Toxicokinetic modeling of 3-OHBaP**

$\frac{\partial}{\partial t}A_{iv}(t)=-ka_{iv}A_{iv}(t)+D_{iv}(t)$, (27)

$\frac{\partial}{\partial t}A_{der}(t)=-ka_{der}A_{der}(t)+D_{der}(t)$, (28)

$\frac{\partial}{\partial t}A_{oral}(t)=-ka_{oral}A_{oral}(t)+D_{oral}(t)$, (29)

$\frac{\partial}{\partial t}A_{inh}(t)=-ka_{inh}A_{inh}(t)+D_{inh}(t)$, (30)

$\frac{\partial}{\partial t}B(t)=-k_{b}B(t)+ka_{iv}A_{iv}(t)+ka_{der}A_{der}(t)+ka_{oral}A_{oral}(t)+ka_{inh}A_{inh}(t)$, (31)

$\frac{\partial}{\partial t}U_{inh}(t)=\alpha k_{b}B(t)$. (32)
